# Aggregation-Induced Emission-Fluorescent-Microsphere-Based Lateral Flow Immunoassay for Highly Sensitive Detection of Capsaicinoids

**DOI:** 10.3390/foods14213634

**Published:** 2025-10-24

**Authors:** Yuchen Bai, Xinyue Han, Yang Yang, Zhanhui Wang, Fubin Qiu

**Affiliations:** 1 MOE Key Laboratory of Coal Environmental Pathogenicity and Prevention, Department of Nutrition and Food Hygiene, College of Public Health, Shanxi Medical University, Taiyuan 030001, China; xy02032527@163.com (X.H.); 15939487708@163.com (Y.Y.); 2 State Key Laboratory of Veterinary Public Health and Safety, College of Veterinary Medicine, China Agricultural University, Beijing 100193, China; wangzhanhui@cau.edu.cn

**Keywords:** aggregation-induced emission, lateral flow immunoassay, capsaicinoids, waste oil, sensitive detection

## Abstract

Capsaicinoids (CPCs) are regarded as a typical marker of waste oil, which has emerged as a serious food safety issue in developing countries, necessitating the development of rapid, sensitive, and specific detection methods. In this study, a novel hapten was synthesized to generate a high-affinity monoclonal antibody (mAb) targeting CPCs. Subsequently, aggregation-induced emission fluorescent microspheres (AIEFMs), known for their superior fluorescence intensity, were utilized as an enhanced probe to develop a lateral flow immunoassay (LFIA) based on mAb 8B4 for CPC detection. For comparison, a traditional gold nanoparticle (AuNP)-LFIA was also constructed using the corresponding mAb. The AIEFM-LFIA demonstrated a limit of detection (LOD) of 0.33 µg/kg for CPCs in edible oil samples, which is 4.21 times lower than the LOD of 1.39 µg/kg achieved by the AuNP-LFIA. And the assay effectively identified three additional CPCs, with LODs ranging from 0.26 to 0.99 µg/kg, while exhibiting minimal cross-reactivity with CPC analogs, indicating high specificity. The recovery rates of the AIEFM-LFIA in oil samples ranged from 75.0% to 106.0%, with coefficients of variation ≤ 8.3%, exhibiting excellent accuracy and precision. Furthermore, the results of the AIEFM-LFIA demonstrated a strong degree of correlation with liquid chromatography–tandem mass spectrometry, with a correlation coefficient (R^2^) of 0.978. Consequently, the developed AIEFM-LFIA shows significant promise as a rapid, sensitive, specific, and reliable method for detecting CPCs in oil samples.

## 1. Introduction

Waste oil, generally referring to a variety of abandoned inferior inedible oils, primarily consists of waste cooking oils recycled from drains and collected from food waste. The adulteration of edible oil with refined waste oil has been one of the most concerning problems in the current food safety landscape of developing countries [1,2,3,4]. The heavy metals and pathogenic bacteria contained in waste oil can adversely impact human health, and long-term intake can lead to liver, heart, and lung damage, and even cancer [5,6]. Therefore, it is imperative to identify representative markers and develop reliable methods for the identification of waste oil. Numerous efforts have been devoted to differentiating oils based on the assessment of the values of iodine, peroxide, and acid in edible oils or the detection of foreign contaminants, such as benzopyrene, aflatoxin, and plasticizers [7,8,9,10,11,12]. Nevertheless, these inherent components can only reflect the quality of oil, which is inadequate for definitively determining whether the oil originates from recycled waste oil. On the other hand, the concentrations of the above-mentioned foreign contaminants in waste oil are reduced to very low levels by treatment processes, thereby impeding their detection [4]. Therefore, a gold standard based on representative markers and reliable methods for discriminating waste oil is urgently required.

Capsaicinoids (CPCs), including natural CPCs and N-vanillylnonanamide (N-V), are the main chemicals responsible for pungency, typically consisting of a vanillyl amide and an alkane chain moiety. The major components of natural CPCs are capsaicin (CPC), dihydrocapsaicin (DCPC), and nordihydrocapsaicin (NDCPC), at proportions of 69%, 22%, and 7%, respectively [13,14,15]. CPCs can stably exist in cooking oils owing to their strong lipophilicity and stable physicochemical properties, and it is difficult to remove all of them during the entire process of waste oil refining. For these reasons, CPCs have been validated as the most representative specific marker for the identification of waste oil in China [16,17,18].

Currently, the detection strategies for CPCs in edible oils predominantly rely on instrumental analytical techniques such as high-performance liquid chromatography (HPLC) and liquid chromatography–tandem mass spectrometry (LC-MS/MS) [13,17,18]. Although these methods possess the significant merits of high sensitivity and high accuracy, allowing for precise quantification of CPCs, they also have unavoidable drawbacks: the sample pretreatment process is cumbersome and complex, failing to meet the demand for rapid detection; the instruments used are sophisticated and expensive, with strict requirements for the operating environment; and the cost of consumables remains high, which greatly limits their wide application in routine detection and on-site screening [19].

In contrast, immunoassay techniques present notable advantages, including rapidity, simplicity, and cost-effectiveness, providing a highly promising alternative for on-site screening of CPCs [20,21]. To date, a limited number of immunoassay methods for CPC determination have been reported [22,23,24,25,26,27,28,29,30,31]. Among these, the lateral flow immunoassay (LFIA) is more suitable for immediate on-site and portable detection. However, the limits of detection (LODs)—ranging from 1 to 20 μg/kg for CPCs in oil samples—achieved by these LFIAs utilizing traditional probes, such as colloidal gold nanoparticles (AuNPs), quantum dots, and time-resolved microspheres, are not sensitive enough to meet detection requirements [23,25,26,30] (Table S1). The reasons for this situation are probably (1) insufficient antibody affinity and (2) weak readout signals, leading to low signal-to-noise ratios. Thus, enhancing antibody affinity and constructing labeling probes with higher signals are crucial for improving the performance of LFIAs for CPCs.

In comparison to colorimetric probes based on horseradish peroxidase and AuNPs, fluorescent probes, such as quantum dots, carbon dots, and time-resolved microspheres, offer higher detection sensitivity and quantitative accuracy owing to high signal-to-noise ratios and low background interference [32,33,34]. Unfortunately, these conventional fluorescent materials frequently encounter aggregation-caused quenching (ACQ) at high concentrations or when aggregated, leading to diminished emission and decreased detection sensitivity in LFIAs [35]. AIEgens (aggregation-induced emission luminogens) provide intense fluorescence upon aggregation, thus overcoming the ACQ limitations of traditional fluorophores and showing promise in immunoassays [36,37,38].

In the present study, a novel hapten of CPCs was synthesized and used to produce a monoclonal antibody (mAb), and AIE fluorescent microspheres (AIEFMs) with superior fluorescence intensity were used as enhanced fluorescent probes to construct a competitive LFIA for sensitive CPC detection. In addition, an LFIA using AuNPs as labeling probes based on the same mAb was also developed for comparison. We then systematically evaluated the potential applications of AIEFMs in LFIAs, focusing on sensitivity, accuracy, specificity, and reliability. In summary, our research showcases a portable, fast, and sensitive LFIA platform, providing an innovative method for the precise detection of CPCs in edible oil samples.

## 2. Materials and Methods

### 2.1. Materials and Reagents

AuNPs were purchased from Invitrogen Corporation (New York, NY, USA). The AIEFMs (carboxyl-functionalized, red-emitting) were purchased from the Institute of Aggregation-Induced Emission, South China University of Technology (Guangzhou, Guangdong, China). Capsaicin (CPC), dihydrocapsaicin (DCPC), nordihydrocapsaicin (NDCPC), and N-vanillylnonanamide (N-V) were obtained from J&K (Shanghai, China). Methyl dopamine hydrochloride, vanillyl amine hydrochloride, and 2-amino-3-(4-hydroxy-3-methoxyphenyl) propionic acid were supplied by Shanghai Bide Pharmaceutical Technology Co., Ltd. (Shanghai, China). The nitrocellulose membrane (NC membrane, UniSart CN 95) was procured from Sartorius AG (Göttingen, Germany). The sample pad (RB65), PVC backplate, absorbent pad, and goat anti-mouse IgG antibody were provided by Shanghai Jinbiao Biotechnology Co., Ltd. (Shanghai, China). Ninety-six-well microtiter plates were purchased from Costar (Cambridge, MA, USA), and bovine serum albumin (BSA) was obtained from Amresco (Solon, OH, USA). Morpholinoethanesulfonic acid (MES), 1-(3-dimethylaminopropyl)-3-ethylcarbodiimide hydrochloride (EDC), and ProClin 300 were acquired from Sigma (St. Louis, MO, USA), while N-hydroxysuccinimide (NHS) was purchased from Shanghai Aladdin Biochemical Technology Co., Ltd. (Shanghai, China). Hemocyanin from Megathura crenulata (KLH), bovine serum albumin (BSA), complete and incomplete Freund’s adjuvant, poly(ethylene glycol), and fetal calf serum were acquired from Sigma-Aldrich (St. Louis, MO, USA). Other conventional reagents were domestic analytical grade and biochemical reagents. Details about the buffers and solutions employed in this study are available in Supplementary Information.

The Milli-Q ultrapure water system was purchased from Millipore (Billerica, MA, USA). The intelligent ultrasonic cleaner was obtained from Shanghai Zhixin Instrument Co., Ltd. (Shanghai, China). The strip cutter, blast drying oven, and ZYX three-dimensional spraying machine were supplied by Shanghai Jinbiao Biotechnology Co., Ltd. (Shanghai, China). The lateral flow chromatography quantitative reader was procured from QIAGEN (Hilden, Germany). The ultra-micro-spectrophotometer and high-speed refrigerated centrifuge were obtained from Thermo (Waltham, MA, USA). The heating magnetic stirrer was purchased from Shanghai Zhenrong Scientific Instrument Co., Ltd. (Shanghai, China). The constant-temperature incubator was supplied by Shanghai Precision Test Equipment Co., Ltd. (Shanghai, China).

### 2.2. Synthesis of Hapten and Preparation of mAb

**Figure 1:**
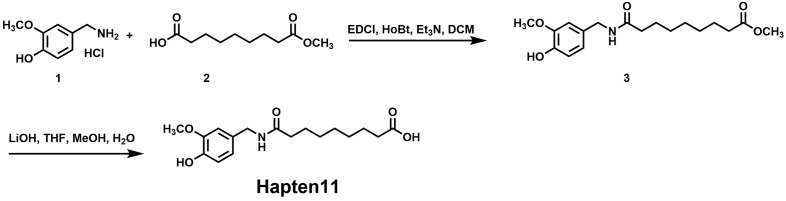
The synthetic route of hapten 11.

As shown in Figure 1, 9-methoxy-9-oxononanoic acid (4.80 g, 23.74 mmol) was dissolved in dichloromethane (DCM, 120 mL), followed by the addition of HoBt (3.53 g, 26.00 mmol). The mixture became turbid and was stirred for 15 min. 4-(Aminomethyl)-2-methoxyphenol hydrochloride (1, 4.50 g, 23.74 mmol) was added, followed by the addition of Et_3_N (12 mL, 90.00 mmol). Then, 1-ethyl-3-(3-dimethylaminopropyl)carbodiimide hydrochloride (EDCl, 5.94 g, 31.00 mmol) was added. The mixture was stirred at RT for 24 h. DCM (120 mL) was added to the reaction mixture. The mixture was washed with water (200 mL × 2) and brine (200 mL), then dried over anhydrous sodium sulfate. Removal of the solvent under vacuum gave a viscous oil (8.00 g) as the crude product. The crude product was subjected to silica gel column chromatography (PE: EA = 1:1) to give a colorless oil (3.78 g, 47%) as a pure compound. The compound (3.78 g, 11.20 mmol) was dissolved in MeOH (30 mL) and tetrahydrofuran (THF, 30 mL), followed by the addition of LiOH hydrate (4.20 g, 100.00 mmol) in water (30 mL). The mixture was stirred at RT for 16 h. Then, 3 NHCl (35 mL) was added to the reaction mixture to adjust the pH to around 4~5. The mixture was concentrated under vacuum to remove MeOH and THF. The residue was poured into water (30 mL). The mixture was extracted with DCM (60 mL × 3). The combined organic layers were washed with water (60 mL) and dried over anhydrous sodium sulfate. Removal of the solvent under vacuum gave the desired pure target compound as hapten 11 (2.00 g, 55%). The chemical structure of the hapten was determined by nuclear magnetic resonance spectrometry (NMRS; DRX-300 from Bruker, Billerica, MA, USA) and high-resolution mass spectrometry (HRMS; Agilent Technologies, Santa Clara, CA, USA). 1H NMR (400 MHz, DMSO-d6) δ 1.24 (s, br, 6H), 1.46-1.54 (m, 4H), 2.11 (t, J = 7.2 Hz, 2H), 2.19 (t, J = 7.6 Hz, 2H), 3.73 (s, 3H), 4.14 (d, J = 6.0 Hz, 2H), 6.63 (dd, J = 8.0, 2.0 Hz, 1H), 6.70 (d, J = 8.0 Hz, 1H), 6.80 (d, J = 2.0 Hz, 1H), 8.17 (t, J = 6.0 Hz, 1H), 8.89 (s, br, 1H), 12.02 (s, br, 1H). 13C NMR (100 MHz, DMSO-d6) δ 24.46, 25.30, 28.45, 28.49, 28.52, 33.64, 35.33, 41.79, 55.45, 111.56, 115.13, 119.63, 130.47, 145.30, 147.36, 171.93, 174.49.

The immunogen and coated antigen were prepared by the active-ester method as described in the Supplementary Materials. Then, eight female BALB/c mice (8 weeks old) were allocated to each immunogen and immunized following the protocol previously reported by our group [24]. Specifically, the initial injection contained 100 μg of immunogen emulsified in complete Freund’s adjuvant, which was followed by three subcutaneous injections of 100 μg immunogen mixed with incomplete Freund’s adjuvant. After the fourth immunization, mice showing the strongest antibody affinity were euthanized to facilitate subsequent cell fusion, with the fusion procedures performed as described in the Supplementary Materials [24]. Hybridoma cells capable of producing antibodies were screened, followed by subcloning via the limiting dilution method. Clones that exhibited high inhibition rates were subcloned three times before being used for ascites production. The affinity of mAb was characterized by icELISA as described in the Supplementary Materials. The standard curves were fitted using the following equation in Origin 7.5 software:

Y = (A − B)/[1 + (X/C)^D^] + B (1)

where A and B correspond to the responses at the high and low asymptotes of the curve, respectively. C represents the concentration of the target that leads to 50% inhibition, D is the slope at the sigmoid’s inflection point, and X is the calibration concentration.

### 2.3. Preparation of AIEFM-mAb and AuNP-mAb

#### 2.3.1. Preparation of AIEFM-mAb

A total of 0.1 mg of AIEFMs was dissolved in 100 μL of activation solution. NHS and EDC were added sequentially, and the mixture was centrifuged at 12,000 r/min for 15 min. The supernatant was discarded, and the precipitate was resuspended in 100 μL of coupling buffer and subjected to ultrasonication for 2 min. Then, the diluted mAb was added, the mixture was centrifuged, and the precipitation was resuspended in 400 μL of blocking solution, shaken at 120 r/min for 2 h, and centrifuged again. The final precipitate was resuspended in 200 μL of fluorescent microsphere reconstitution buffer and stored at 4 °C.

#### 2.3.2. Preparation of AuNP-mAb

The AuNP solution’s pH was adjusted using 0.1 M K_2_CO_3_, followed by the rapid addition and mixing of the diluted mAb. A 20 μL volume of 20% BSA solution (*w*/*v*) was added after a 15 min reaction at room temperature and then incubated for an additional 15 min. The mixture was centrifuged at 10,000 r/min for 15 min. The supernatant was discarded, and the precipitate was resuspended in 0.02 M PBS (containing 1% BSA, pH 7.4) and stored at 4 °C.

Transmission electron microscopy (TEM) was used to characterize the morphology and particle size of the detection probes, while a Malvern laser particle size analyzer was employed to assess their hydrodynamic particle size.

### 2.4. Fabrication and Optimization of LFIA Test Strips

AIEFMs and AuNPs were prepared as labels for constructing the LFIA. The test strip consisted of five components: the sample pad, conjugate pad, NC membrane, absorbent pad, and PVC backing plate. Prior to assembly, the sample pad was immersed in 0.01 M PBS (pH 7.4) containing 0.2% Tween-20, 1.0% sucrose, and 1.0% BSA to decrease matrix interference, followed by drying at 37 °C. The NC membrane was coated with CPC-BSA conjugates as coated antigen and goat anti-mouse IgG antibody to create the test (T) line and control (C) line, respectively, at a rate of 3 µL/cm using a XYZ three-dimensional gold spraying device, followed by drying at 37 °C overnight. Next, the conjugate pad was sprayed with the detection probe at a rate of 3 µL/cm and then dried in a 37 °C oven for 90 min. Ultimately, the sample pad, conjugate pad, dried NC membrane, and absorbent pad were stacked with a 2 mm overlap on a PVC backing plate. After assembly, the card was sliced into 4 mm wide strips and stored at room temperature.

To develop a highly sensitive AIEFM-LFIA for CPC detection, various parameters were systematically optimized, including buffer pH, monoclonal antibody (mAb) dosage, AIEFM probe dosage, and coated antigen concentration, by comparing T/C values and inhibition rates. Specifically, the buffer pH was evaluated at levels of 5.0, 6.0, 7.0, 8.0, and 9.0 while maintaining constant conditions for the mAb dosage (10 μg), AIEFM probe dosage (2.0 μL), and coated antigen concentration (0.5 mg/mL). The mAb dosage was assessed at 10 μg, 20 μg, 30 μg, 40 μg, and 50 μg, with fixed conditions of pH 7.0, AIEFM probe dosage (2.0 μL), and coated antigen concentration (0.5 mg/mL). Similarly, the AIEFM probe dosage was tested at 1.0 μL, 2.0 μL, 3.0 μL, 4.0 μL, and 5.0 μL under constant conditions of pH 7.0, mAb dosage (10 μg), and coated antigen concentration (0.5 mg/mL). Furthermore, the coated antigen concentration was varied at 0.2 mg/mL, 0.3 mg/mL, 0.4 mg/mL, 0.5 mg/mL, and 0.6 mg/mL, with the buffer pH set at 7.0, mAb dosage at 10 μg, and AIEFM probe dosage at 2.0 μL. For the construction of AuNP-LFIA strips, the K_2_CO_3_ dosage (0 μL, 10 μL, 20 μL, 30 μL, and 40 μL), the dosage of mAb (10 μg, 20 μg, 30 μg, 40 μg, and 50 μg), the dosage of AuNP probes (1.0 µL, 2.0 µL, 3.0 µL, 4.0 µL, and 5.0 µL), and the concentration of coated antigen (0.2 mg/mL, 0.3 mg/mL, 0.4 mg/mL, 0.5 mg/mL, and 0.6 mg/mL) were optimized.

### 2.5. Oil Sample Preparation

A collection of ten different kinds of soybean oil, maize oil, peanut oil, canola oil, olive oil, sunflower oil, and blend oil was obtained from markets. After adding standard solutions of CPC, DCPC, NDCPC, and N-V at different concentrations to 10.0 g oil samples, the samples were extracted twice with 5 mL of methanol. After centrifugation, the organic layer on top was separated from the oil layer. After drying the 2 mL organic layer with nitrogen gas, it was reconstituted with 1 mL of 20% methanol in 0.01 M PBS. Finally, 50 μL of the reconstitution solution was taken and mixed with 950 μL of 0.01 M PBS for LFIA.

### 2.6. Validation of AIEFM-LFIA

To verify the superiority of the proposed assay, the sensitivity, accuracy, specificity, and reliability of AIEFM-LFIA were assessed.

#### 2.6.1. Sensitivity

The calculated LOD (cLOD) was established as the average value of the blank plus the triplex value of the standard deviation from the recorded values of a total of 40 blank samples. And the visual LOD (vLOD), defined as the lowest concentration at which the T-line disappears, was determined by observing T-line visibility on test strips.

#### 2.6.2. Specificity of Assay

One-gram portions of blank edible oil samples were weighed and spiked with three CPCs (DCPC, NDCPC, N-V) and CPC analogs [methyl dopamine hydrochloride, vanillyl amine hydrochloride, 2-amino-3-(4-hydroxy-3-methoxyphenyl) propanoic acid, guaiacol, 4-methylguaiacol, 4-ethylguaiacol, acetovanillone, vanillyl alcohol, vanillin oxime, 4-(2-aminoethyl)-2-methoxyphenol, and vanillyl amine]. Each standard was spiked at a concentration of 2 μg/kg. The specificity was assessed by a cross-reactivity experiment. The cross-reactivity (CR%) was calculated using the following formula:

CR (%) = (Measured value/Spiked concentration) × 100%(2)

#### 2.6.3. Accuracy

One-gram portions of blank edible oil samples were weighed, and experiments were repeated three times. Each sample was spiked with a CPC, DCPC, NDCPC, or N-V standard at a concentration of 1.0, 1.6, or 2.4 μg/kg. After preconditioning, the spiked samples were analyzed using the established AIEFM-LFIA. The accuracy and precision were assessed through recovery experiments. The recovery rates and the coefficients of variation (CVs) were calculated using the following formulas:

Recovery (%) = (Measured value/Spiked value) × 100%(3)

CV (%) = (Standard deviation/Mean value) × 100% (4)

#### 2.6.4. Reliability

The 20 oil samples containing different concentrations of CPCs were simultaneously analyzed using the developed AIEFM-LFIA and LC-MS/MS to verify the reliability of the proposed method. The detection of CPCs by LC-MS/MS was performed according to the BJS 201801, Determination of Capsaicin in Edible Oils.

## 3. Results

### 3.1. Preparation and Identification of Hapten and mAb

In this study, we synthesized a novel hapten named hapten11 by fully protecting and emphasizing the main functional groups of the CPCs, i.e., alkane chains and vanillyl amide moieties, to ensure complete exposure to the immune system. The synthetic hapten11 was purified and identified by NMRS and HRMS (Figure S1), confirming the successful synthesis of hapten11. Hapten11 was employed to immunize mice for mAb production. After screening and cloning, the most sensitive mAb 8B4 with the highest dilution (1/1.8 × 10^5^) was obtained. mAb 8B4 and the coated antigen were diluted to various concentrations, and the affinity of the mAb (IC_50_ = 1.13 ng/mL, Figure S2) was obtained. Furthermore, the isotype of 8B4 was IgG_1_, whereas the type of light chain was Lambda. Overall, mAb 8B4 showed high titer and affinity, suggesting that the newly designed hapten11 effectively induced a robust immune response.

### 3.2. Characterization of AIEFMs, AIEFM-mAb, AuNPs, and AuNP-mAb

**Figure 2:**
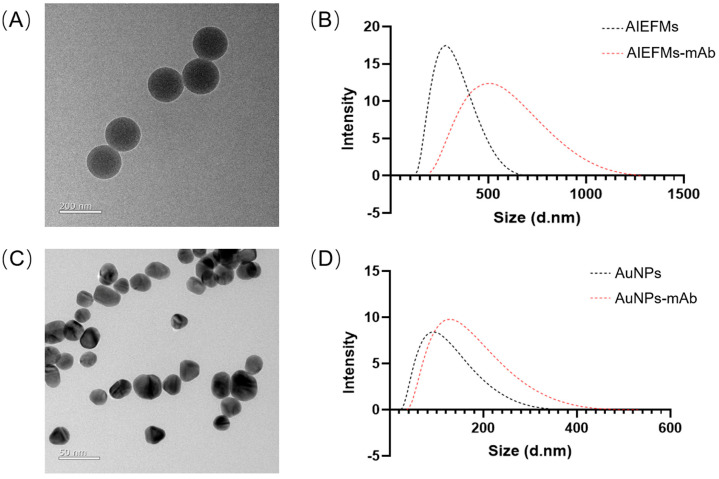
Characterization of AIEFMs, AIEFM-mAb, AuNPs, and AuNP-mAb. (**A**) TEM image of AIEFMs. (**B**) Hydrated particle sizes of AIEFMs and AIEFM-mAb. (**C**) TEM image of AuNPs. (**D**) Hydrated particle sizes of AuNPs and AuNP-mAb.

In the present study, AIEFMs were used to label mAb 8B4 as probes for CPC detection. For comparison, AuNP-mAb was employed as a detection probe to verify the superiority of AIEFMs in assay sensitivity. As shown in Figure 2A,B, the transmission electron microscope (TEM) images revealed that the AIEFMs possessed nearly spherical shapes and a consistent particle size distribution with an average diameter of 139.4 nm. Dynamic light scattering (DLS) analysis was performed to assess the hydrodynamic particle size of AIEFMs and AIEFM-mAb (Figure 2B). The hydrodynamic diameter of AIEFMs–mAb was measured at 504.3 nm, which is markedly larger than that of the unmodified AIEFMs (291.5 nm), indicating the successful conjugation of mAb to the AIEFMs’ surface. Furthermore, to investigate the effect of phenolics/antioxidants present in edible oils on the fluorescence intensity of AIEFMs [39,40], 0.2 μg/mL tertbutylhydroquinone (TBHQ) and 0.6 μg/mL bisphenol A (BPA) were added to the AIEFM solution and incubated for 15 min. As shown in Figure S3, the fluorescence intensities of the solutions containing TBHQ and BPA did not show significant deviation compared to the control AIEFM solution. This result confirmed the stability of the fluorescence intensity of AIEFMs. And Figure 2C shows a typical TEM image of a 50 nm AuNP. The hydrodynamic particle sizes of AuNPs and AuNP-mAb were determined to be 101.9 nm and 138.1 nm, respectively (Figure 2D). The significant increase in hydrodynamic diameter indicated successful conjugation of mAb to the surface of AuNPs.

### 3.3. Detection Mechanism of AIEFM-LFIA

**Scheme 1:**
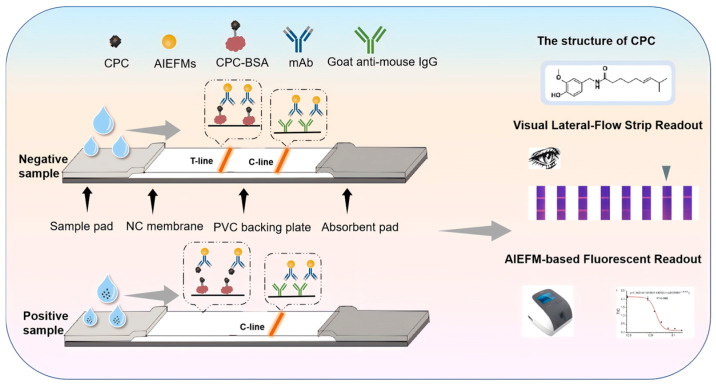
Schematic diagram of AIEFM-LFIA.

For the rapid, simple, accurate, and highly sensitive detection of CPCs, AIEFMs with enhanced fluorescence intensity were proposed to construct an AIEFM-LFIA. Given that CPCs are small molecules with low molecular weight (structure of CPC is shown in Scheme 1), the competitive reaction between a carrier-protein-labeled analyte and the analyte itself for binding to the antibody was applied as the detection mechanism of the LFIA. Specifically, CPC-BSA and goat anti-mouse IgG were immobilized on the NC membrane as the T-line and C-line, respectively. In the absence of CPCs in the sample, AIEFM-mAb flows through the NC membrane and is captured by CPC-BSA on the T-line, while excess probe is sequestered by goat anti-mouse IgG on the C-line, thus producing a bright fluorescent band on both the T- and C-lines. Conversely, in the presence of CPCs in the sample, these compounds compete with CPC-BSA for binding to AIEFM-mAb. Consequently, as the concentration of CPCs in the sample increases, there is a corresponding decrease in the fluorescence intensity of the T-line. This enables quantitative analysis by assessing the ratio of fluorescence intensity between the T-line and the C-line, ensuring a reliable method for CPC quantification.

### 3.4. Optimization of AIEFM-LFIA

**Figure 3:**
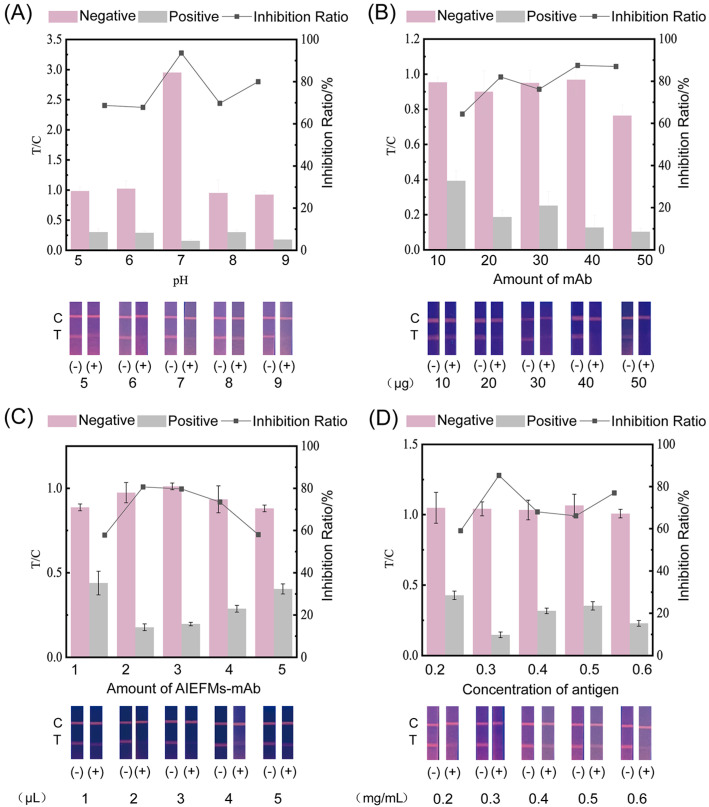
Optimization results of AIEFM-LFIA preparation conditions (*n* = 3). Fluorescence intensity of T-line and C-line, T/C value, and inhibition rates in different (**A**) pH values of the buffer solution, (**B**) antibody dosages, (**C**) probe dosages, (**D**) coated antigen concentrations.

The AIEFM-LFIA’s performance was enhanced by optimizing key parameters, such as buffer system pH, mAb dosage, fluorescent probe, and coated antigen, to achieve higher T/C values and increased inhibition rates. Figure 3A demonstrates that at a pH of 7.0 in the BB buffer, a distinct fluorescent reaction and noticeable fading were observed at a negative T-line and positive T-line, respectively, and the inhibition rate (1 − T/T_0_) reached its highest value in the positive sample, which was identified as the optimal pH for further experiments. As illustrated in Figure 3B, a high 1 − T/T_0_ value of 82.4% was obtained when T_0_ was measured at 6085, indicating that the optimal dosage of mAb conjugated to AIEFMs was 20 μg. The optimal amount of probe used per strip was determined to be 2 μL, as the T/T_0_ value reached its maximum (Figure 3C). In addition, the optimal dosage of coated antigen was found to be 0.3 mg/mL (Figure 3D). To confirm the detection capabilities of the prepared AIEFM-LFIA, a AuNP-LFIA was also constructed for CPC detection using the same mAb and detection antigens. Likewise, several parameters that may influence the performance of the assay were optimized. As depicted in Figure S4A–D, the most effective dosage of K_2_CO_3_ was 40 μL, the best amount of mAb was 20 μg, the optimal probe per strip was 1 μL, and the ideal concentration of coated antigen was 0.2 mg/mL.

### 3.5. Detection Performance of AIEFM-LFIA and AuNP-LFIA in Oil Samples

**Figure 4:**
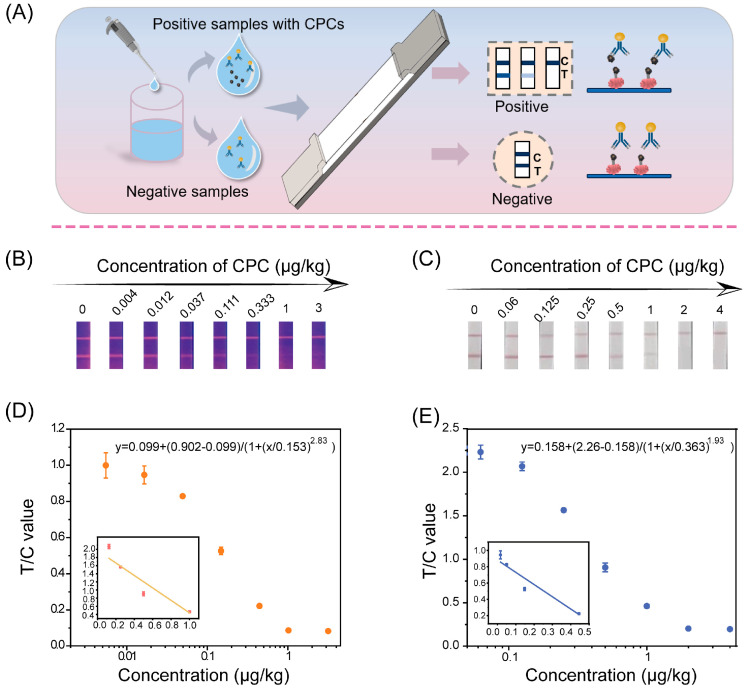
(**A**) Illustration showing the positive and negative results of LFIA. (**B**) Images of test strips obtained from AIEFM-LFIA. (**C**) Images of test strips obtained from AuNP-LFIA. (**D**) Standard curve of AIEFM-LFIA. (**E**) Standard curve of AuNP-LFIA. Insets of (**D**,**E**) show linear range regions of AIEFM-LFIA and AuNP-LFIA.

**Figure 5:**
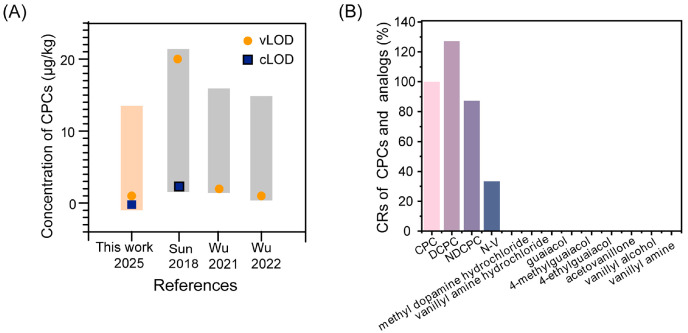
(**A**) VLOD and cLOD in the present work compared with those of published LFIAs for CPC detection [23,25,26]. (**B**) Specificity results of AIEFM-LFIA for detecting CPCs and CPC analogs.

Under the respective optimal reaction conditions, CPC, used as a representative analyte at a series of concentrations, was measured using the AIEFM-LFIA and AuNP-LFIA in diluted oil samples (Figure 4A). As shown in Figure 4B,D, as the CPC concentration increased, the fluorescence (color) intensity of the T-line gradually diminished, demonstrating that the quantitative capabilities of AIEFM-LFIA and AuNP-LFIA can be evaluated through a distinct relationship that depends on concentration. The vLODs of the AIEFM-LFIA and AuNP-LFIA, corresponding to the disappearance of coloration in the analytical zone, were 0.333 µg/kg and 2 µg/kg, respectively. Then, the calibration curves of the AIEFM-LFIA and AuNP-LFIA were constructed by plotting T/C intensity against CPC concentration, defined by the equations y = 0.099 + 0.803/(1 + (x/0153) ^2.83^) (R^2^ = 0.990) and y = 0.158 + 2.012/(1 + (x/0.363) ^1.93^ (R^2^ = 0.983), respectively (Figure 4D,E). The cLOD—established as the average value of the blank plus the triplex value of the standard deviation from the recorded values of a total of 40 blank samples, consisting of 20 samples from each of two batches—of the AIEFM-LFIA was 0.33 µg/kg, which exhibited a 4.21-fold enhancement compared to that of the AuNP-LFIA (1.39 µg/kg). Moreover, as shown in Figure 5A and Table S1, the AIEFM-LFIA exhibited significant advantages in sensitivity compared to other reported LFIAs for CPC detection, including vLOD and cLOD, with 3~60-fold and 7.43-fold improvements, respectively [23,25,26]. These findings suggest that we have effectively developed a sensitive AIEFM-LFIA platform with significant robustness.

The specificity of the AIEFM-LFIA was evaluated by determining cross-reactivity rates (CRs) with the other three CPCs (DCPC, NDCPC, and N-V) and CPC analogs. As shown in Table S2 and Figure 5B, the AIEFM-LFIA developed in this study can recognize three CPCs well, with CRs ranging from 87.2% to 127.2%. Therefore, the LODs of DCPC, NDCPC, and N-V were 0.26, 0.38, and 0.99 µg/kg in oil samples, respectively. In addition, eight CPC analogs, including methyl dopamine hydrochloride, vanillylamine hydrochloride, and others, were also detected by the established assay. Since they naturally occur in various vegetable oils or are used as food additives, they might coexist with CPCs during waste oil processing, potentially leading to false-positive outcomes. Negligible CRs with CPC analogs are observed in Table S2. Notably, these results demonstrate the high specificity of the AIEFM-LFIA for detecting CPCs.

### 3.6. The Accuracy and Precision of the AIEFM-LFIA

The accuracy and precision of the AIEFM-LFIA for the detection of four CPCs were evaluated by testing spiked positive samples with CPC concentrations of 1.00, 1.60, and 2.40 μg/kg. Each concentration was tested five times, and the corresponding recoveries and coefficients of variation (CVs) were recorded. As shown in Table 1, the average recovery rates ranged from 75.0% to 106.0%, and the CVs ranged from 2.1% to 8.3%. Therefore, the AIEFM-LFIA developed in this study exhibited high accuracy and precision, fully satisfying the requirements for practical detection applications.

### 3.7. The Stability and Reliability of the AIEFM-LFIA

To assess the thermal stability of the prepared AIEFM-LFIA test strip, a short-term accelerated stability evaluation was conducted in an oven at 60 °C. Notably, following the aging process, the variations in the fluorescence intensity of the T-line of the AIEFM-LFIA test strip consistently remained below 24.7% (Figure S5). The result allowed the prediction that the test strip can be stored at room temperature for 6~9 months, further confirming the favorable thermal stability of the AIEFM-LFIA test strip [41,42].

**Figure 6:**
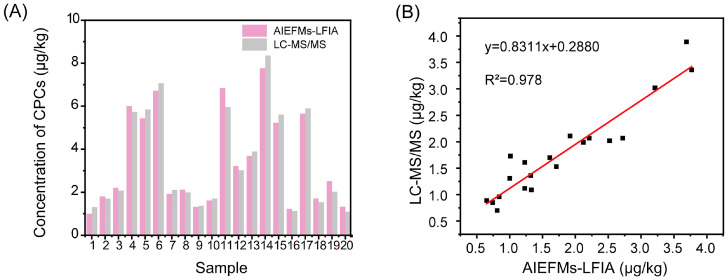
(**A**) Comparison of detection results between AIEFM-LFIA with LC/MS-MS in 20 oil samples (*n* = 3). (**B**) Correlation analysis of AIEFM-LFIA and LC/MS-MS.

Furthermore, twenty edible oil samples, spiked with varying concentrations of CPCs, were randomly selected, and each sample was analyzed using the AIEFM-LFIA developed in this study, as well as the LC-MS/MS method specified in BJS 201801, Determination of Capsaicin in Edible Oils. As shown in Figure 6A and Table S3, the CPC contents detected by the LFIA ranged from 0.81 to 3.77 μg/kg, whereas those measured by LC-MS/MS ranged from 0.70 to 3.89 μg/kg. A correlation analysis of the results from the two methods revealed a high degree of consistency, with a correlation coefficient exceeding 0.978 (Figure 6B). Notably, the AIEFM-LFIA requires only 20 min for detection, is low-cost, and does not require professional expertise for operation. Collectively, the established AIEFM-LFIA enables efficient, high-throughput screening of samples with both high reliability and practical applicability.

## 4. Conclusions

Herein, we have developed a highly sensitive, rapid, and specific AIEFM-LFIA for CPC detection in edible oils based on mAb 8B4. The assay addresses the limitations of traditional LFIA methods, which often suffer from low sensitivity and limited applicability for CPC detection. The key breakthrough is the preparation of high-affinity mAb 8B4 and the application of AIEFMs as signal probes, which differ from conventional probes such as AuNPs and quantum dots. Benefiting from AIEFMs’ intense fluorescence and low matrix interference, the AIEFM-LFIA achieved sensitive CPC detection with a LOD of 0.33 μg/kg—a 4.21-fold enhancement over traditional AuNP-LFIA and a 3~60-fold improvement compared to previous LFIA reports [23,25,26,30]. Notably, the AIEFM-LFIA could simultaneously detect four CPCs (CPC, DCPC, NDCPC, and N-V) without CRs with other CPC analogs, demonstrating excellent specificity. The accuracy and precision of the AIEFM-LFIA were confirmed in oil samples, with recovery rates varying from 75.0% to 106.0% and coefficients of variation ≤ 8.3%. Importantly, the detection results for edible oil samples obtained using the AIEFM-LFIA were consistent with those from LC-MS/MS, confirming the assay’s reliability. Furthermore, the materials employed in our study, including AIEFMs and test strips, are cost-effective, and mAb 8B4 was produced by our laboratory, enabling large-scale production of ascites. Additionally, the preparation process is amenable to automation, thus exhibiting considerable potential for industrial-scale production. Collectively, AIEFMs showed great promise as potential probes for LFIAs, offering a robust strategy for ultrasensitive point-of-care testing.

## Figures and Tables

**Table 1:** Recoveries and CVs of AIEFM-LFIA (n = 5).

Analyte	Amount Spiked (μg/kg)	Recovery (%)	CV (%)
CPC	1.00	92.0	4.0
	1.60	88.0	2.3
	2.40	91.0	6.8
DCPC	1.00	90.0	2.6
	1.60	106.0	2.1
	2.40	103.0	4.2
NDCPC	1.00	81.0	3.4
	1.60	106.0	7.9
	2.40	87.0	4.7
N-V	1.00	75.0	8.3
	1.60	99.0	6.3
	2.40	88.0	7.2

## Data Availability

The original contributions presented in this study are included in the article/supplementary material. Further inquiries can be directed to the corresponding author.

## Supplementary Materials

The following supporting information can be downloaded at https://www.mdpi.com/article/10.3390/foods14213634/s1: Figure S1: The mass spectra and ^1^H NMR spectra of hapten11. Figure S2: Calibration curve of icELISA based on mAb 8B4 for CPC. Figure S3: The fluorescence values of the solutions of (A) AIEFMs; (B) with 0.2 μg/mL TBHO; and (C) 0.6 μg/mL with BPA. Figure S4: Optimization results of AuNP-LFIA preparation conditions. Fluorescence intensity of T-line and C-line in different (A) K_2_CO_3_, (B) antibody, and (C) probe dosages and (D) coated antigen concentrations. Figure S5: Evaluation of thermal stability for AIEFM-LFIA test strip during a 7-day aging period at 60 °C. The error bars represent standard deviations (n = 3). Table S1: Reported LFIAs for the determination of CPCs in the literature; Table S2: The LODs and CRs of AIEFM-LFIA with four CPCs and eight analogs; Table S3: Comparison of the detection results between AIEFM-LFIA with LC/MS-MS in 20 oil samples (n = 3).

## Abbreviations

The following abbreviations are used in this manuscript:

CPCs	Capsaicinoids
AIEFMs	Aggregation-induced emission fluorescent microspheres
LFIA	Lateral flow immunoassay
AuNPs	Gold nanoparticle
LOD	Limit of detection
R^2^	Correlation coefficient
CPC	Capsaicin
DCPC	Dihydrocapsaicin
NDCPC	Nordihydrocapsaicin
HPLC	High-performance liquid chromatography
LC-MS/MS	Liquid chromatography–tandem mass spectrometry
N-V	N-vanillylnonanamide
BSA	Bovine serum albumin
MES	Morpholinoethanesulfonic acid
EDC	1-(3-Dimethylaminopropyl)-3-ethylcarbodiimide hydrochloride
NHS	N-hydroxysuccinimide
mAb	Monoclonal antibody
TEM	Transmission electron microscopy
cLOD	Calculated LOD
vLOD	Visual LOD
CR%	Cross-reactivity
DLS	Dynamic light scattering
CVs	Coefficients of variation
