# Supplementary material for: Aggregation-Induced Emission-Fluorescent-Microsphere-Based Lateral Flow Immunoassay for Highly Sensitive Detection of Capsaicinoids
Source: Foods. 2025 Oct 24;14(21):3634. doi: 10.3390/foods14213634 (PMC12608905; doi:10.3390/foods14213634)
Supplement: Supplementary file 1 [file foods-14-03634-s001.zip › foods-3894400-supplementary.pdf]

foods

Supplementary Information

Aggregation-Induced Emission-Fluorescent-Microsphere-Based Lateral Flow Immunoassay for Highly Sensitive Detection of Capsaicinoids

Yuchen Bai [1,*], Xinyue Han [1], Yang Yang [1], Zhanhui Wang [2] and Fubin Qiu [1,*]

[1] MOE Key Laboratory of Coal Environmental Pathogenicity and Prevention, Department of Nutrition and Food Hygiene, College of Public Health, Shanxi Medical University, Taiyuan 030001, China; xy02032527@163.com (X.H.); 15939487708@163.com (Y.Y.)
[2] State Key Laboratory of Veterinary Public Health and Safety, College of Veterinary Medicine, China Agricultural University, Beijing 100193, China; wangzhanhui@cau.edu.cn
* Correspondence: baiyuchen@sxmu.edu.cn (Y.B.); fbqiu@sxmu.edu.cn (F.Q.)

Buffers

The common buffer solutions used in the experiment are listed: (1) coating buffer (CB, 0.05 mol/L, carbonate buffer, pH 9.6); (2) activation solution (0.05 mol/L, MES buffer, pH 6.0); (3) coupling buffer (BB, 0.04 mol/L, borate−borax buffer, pH 8.0); (4) blocking solution (5% skim milk powder (w/v)); (5) reconstitution buffer (0.1 mol/L Tris−HCl, 0.5% BSA (w/v), 0.5% Tween−20 (v/v), 5% sucrose (w/v), 0.9% NaCl (w/v), and 0.02% Proclin300 (v/v), pH 8.0); (6) K_2CO_3 solution (0.1 mol/L, pH 12.0); (7) PBS solution (0.01 mol/L, 0.2% Tween−20 (v/v), 1.0% sucrose (w/v), and 1.0% BSA (w/v), pH 7.4); (8) Coating buffer (0.05 mol/L carbonate buffer, pH 9.6); (9) Blocking buffer (2% skim milk powder (w/v); (10) Washing buffer (PBST, PBS buffer with 0.05% Tween-20 (v/v), pH 7.2); (11) Boric acid buffer (0.02 mol/L, pH 9.0, containing 10% DMF); (12) Stopping reagent (2 mol/L H_2SO_4)

1. Synthesis of Immunogen and coated antigen.

The hapten11 was conjugated to a protein (KLH or BSA) by the active-ester method through their active carboxylic acid groups. First, the hapten (0.1 mM) was dissolved in 0.5 mL of DMF, and then NHS (15.0 mg) and DCC (30.0 mg) were added, which was stirred at room temperature overnight. Then, the solution

(activated hapten) was added dropwise into the dissolved KLH solution (0.001 mM KLH in 10 mL of PBS) or BSA solution (0.001 mM BSA in 10 mL of PBS), respectively, and further stirred for 24 h. The conjugates were dialyzed against PBS (pH 7.0) at room temperature for 72 h. The hapten-KLH served as immunogen and the hapten−BSA acted as coated antigens for hapten11.

2. Cell Fusion

The selected mice were injected intraperitoneally with three times the immune dose (300 μg immunogen in 500 μL PBS buffer). Three days later, SP2/0 myeloma cells and splenocyfrom the selected mice were mixed together for cell fusion under the assistance of PEG 1500. The successfully fusion cells were cultured in HAT medium for 7 days, and the cell supernatant was screened using the icELISA.

3. icELISA

The ELISA plates were first coated with coating antigen (100 μL/well) which was diluted with CB buffer and then incubated at 4 ° C for 12 h. The coating solution in the ELISA plates was then discarded. Blocking buffer was then added to the plates (150 μL/well) which were placed in an incubator for 2 h at 37 ° C. The standard solution (50 μ L) of TMP (or other competitors) was added to the wells as well as 50 μ L of diluted mAbs solution. After an incubation of 30 min at 37 ° C, the plates were washed by PBST two times. Goat anti-mouse IgG (HRP labeled) (1:5000, 100 μ L/well) was then added and incubated for 30 min in an incubator. Next, 100 μ L TMB substrate was added into wells and incubated for 15 min at 37 °C after washing. Finally, 2 mol L−1 H_2SO_4 (50 μL/well) was used to stop the enzymatic reaction, and the OD values at 450 nm were measured.

Table S1. Reported LFIAs for the determination of CPCs in the literature

Reference	Immunoassay platform	vLOD (μg/kg)	cLOD (μg/kg)
**Our study**	**LFIA**	**0.333**	**0.07**
Qingqing Yang et al. (2016) [1]	icELISA & TRFICA	/	1.1 & 1.5
**Jiajia Sun et al. (2018) [2]**	**LFIA**	**1.0-2.0**	**/**
Yuchen Bai et al. (2021) [3]	icELISA	/	0.73-3.29
**Qian Wu et al. (2021) [4]**	**LFIA**	**20**	**2.3**
**Yuxiang Wu et al. (2022)** [5]	**LFIA**	**1**	**/**
Jiali Zhang et al. (2022) [6]	icELISA	/	20
Jiali Zhang et al. (2022) [7]	FPIA	/	1.56
Di Yuan et al. (2023) [8]	TRFICA	0.6	/
**Kunying Nie et al. (2024)** [9]	**LFIA**	**>1**	**/**
Zixin Jin et al. (2024) [10]	NISA	/	0.04

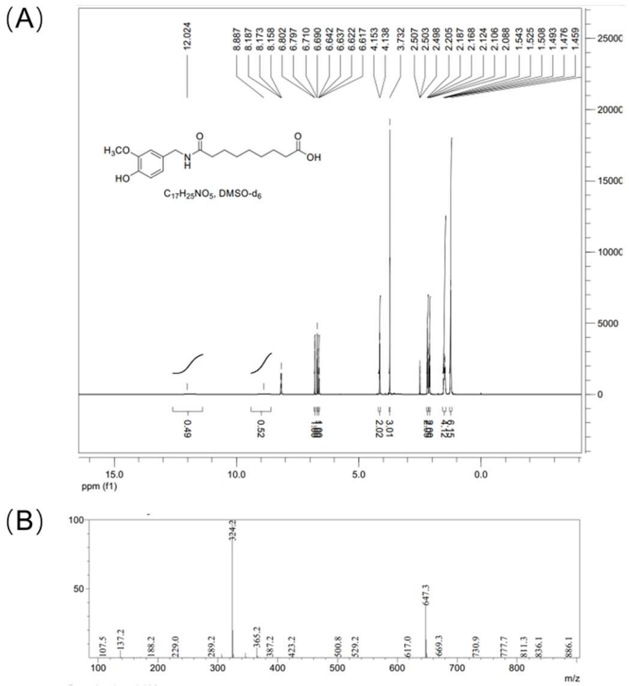

Figure S1. The mass spectra and ^{1}H NMR spectra of hapten11.

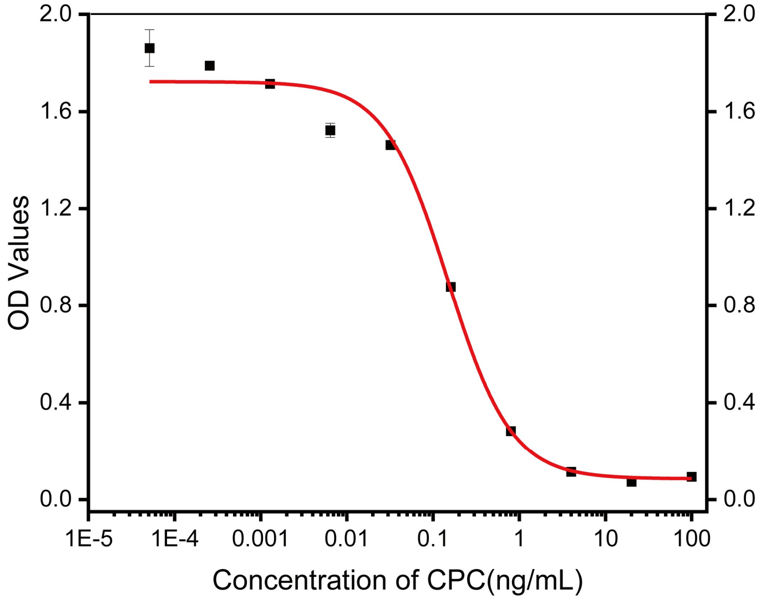

Figure S2. Calibration curve of icELISA based on mAb 8B4 for CPC.

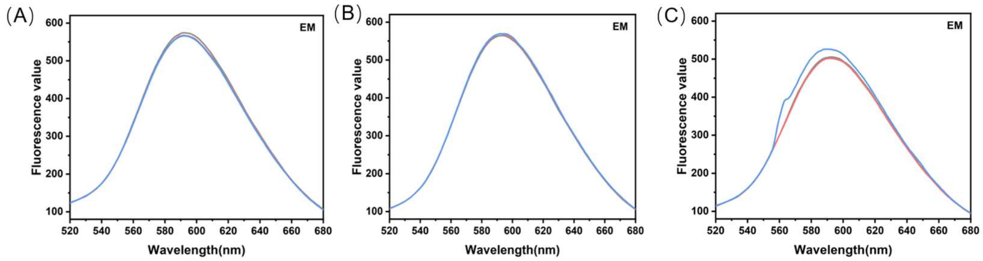

Figure S3. The fluorescence intensities of the solutions of (A) AIEFMs alone; (B) AIEFMs with 0.2 μg/mL TBHO; and (C) AIEFMs with 0.6 μg/mL BPA.

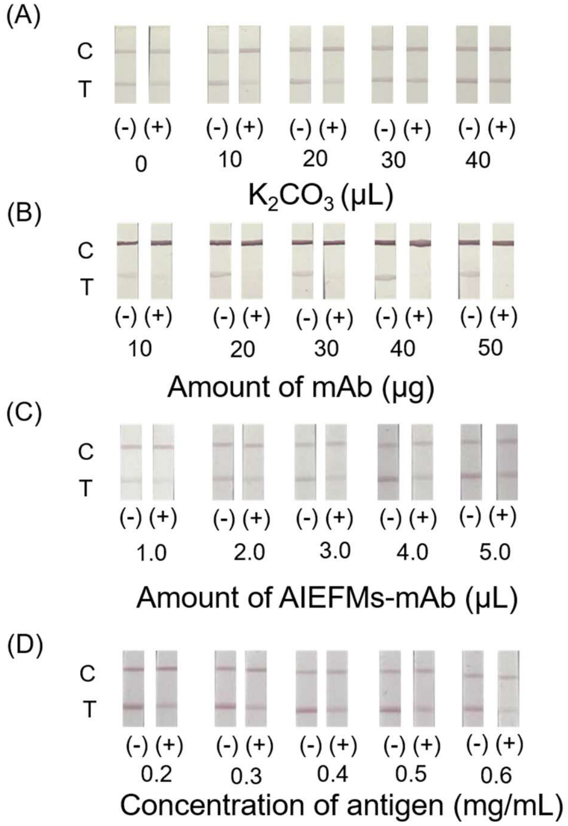

Figure S4. Optimization results of AuNPs-LFIA preparation conditions. Fluorescence intensity of T-line and C-line in different (A) K_2CO_3. (B) Antibody dosage. (C) Probe dosage. (D) Coating antigen concentration

Table S2. The LODs and CRs of AIEFMs-LFIA to four CPCs and eight analogues

Reference standard	LODs (μg/kg)	CRs (%)
CPC	0.33	100.0
DCPC	0.26	127.2
NDCPC	0.38	87.2
N-V	0.99	33.4
methyl dopamine hydrochloride	/	＜0.1
vanillyl amine hydrochloride	/	＜0.1
3-(4-hydroxy-3-methoxyphenyl) alanine	/	＜0.1
guaiacol	/	＜0.1
4-methylguaiacol	/	＜0.1
4-ethylguaiacol	/	＜0.1
acetovanillone	/	＜0.1
vanillyl alcohol	/	＜0.1
vanillyl amine	/	＜0.1

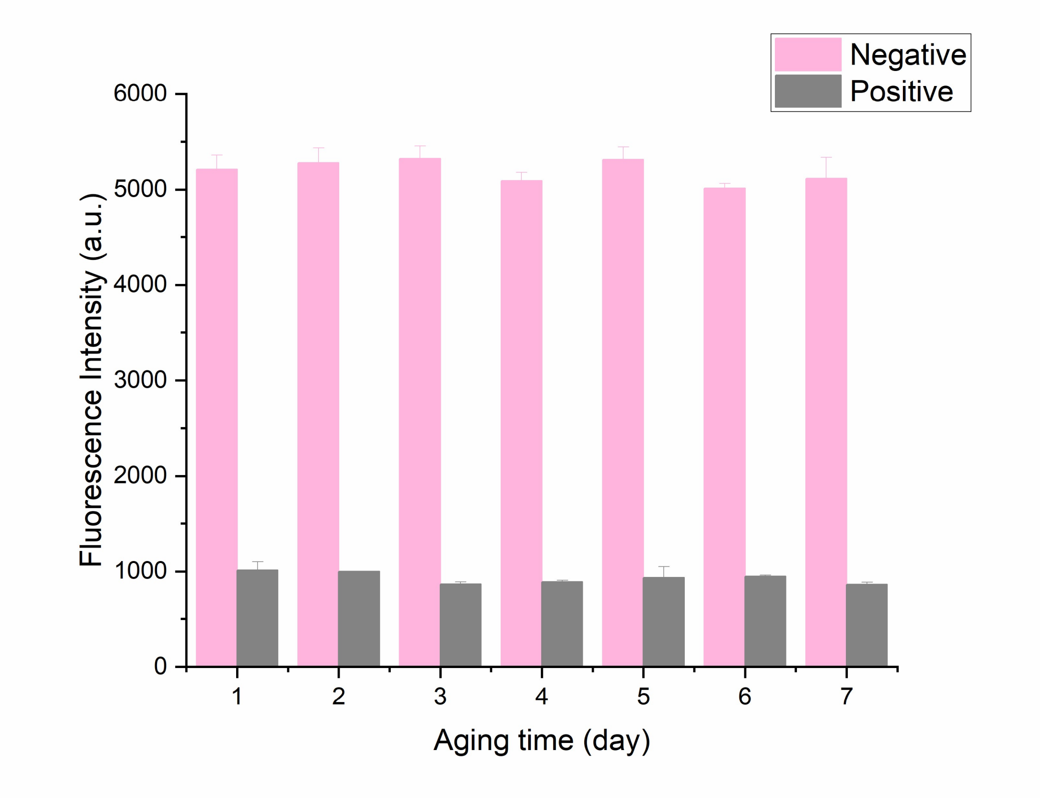

Figure S5. Evaluation of thermal stability for AIEFMs-LFIA test strip during a 7-day aging period at 60 °C. The error bars represent standard deviations (n=3).

Table S3 Comparison of the detection results between AIEFMs-LFIA with the LC/MS-MS in 20 oil samples (n=3).

Samples	AIEFMs-LFIA (μg/kg)	LC-MS/MS (μg/kg)	Samples	AIEFMs-LFIA (μg/kg)	LC-MS/MS (μg/kg)
1	1.00	1.31	11	0.84	0.96
2	0.81	0.70	12	3.21	3.02
3	2.21	2.07	13	3.69	3.89
4	1.01	1.73	14	3.77	3.36
5	0.74	0.85	15	1.23	1.61
6	2.72	2.07	16	1.23	1.12
7	1.92	2.11	17	0.65	0.89
8	2.12	1.99	18	1.71	1.53
9	1.32	1.36	19	2.52	2.02
10	1.61	1.70	20	1.33	1.09

References

1. Yang, Q.; Zhu, J.; Ma, F.; Li, P.; Zhang, L.; Zhang, W.; Ding, X.; Zhang, Q. Quantitative determination of major capsaicinoids in serum by ELISA and time-resolved fluorescent immunoassay based on monoclonal antibodies. *Biosens. Bioelectron.* **2016**, *81*, 229-235, http://dx.doi.org/10.1016/j.bios.2016.02.074

2. Sun, J.; Liu, L.; Song, S.; Cui, G.; Kuang, H. Development of an immunochromatographic strip assay for three major capsaicinoids based on an ultrasensitive monoclonal antibody. *Food Agric. Immunol.* **2018**, *29*, 930-940, http://dx.doi.org/10.1080/09540105.2018.1490394

3. Bai, Y.; Jiang, H.; Zhang, Y.; Dou, L.; Liu, M.; Yu, W.; Wen, K.; Shen, J.; Ke, Y.; Yu, X.; Wang Z. Hydrophobic Moiety of Capsaicinoids Haptens Enhancing Antibody Performance in Immunoassay: Evidence from Computational Chemistry and Molecular Recognition. *J. Agric. Food Chem.* **2021**, *69*, 9957-9967, http://dx.doi.org/10.1021/acs.jafc.1c03657

4. Wu, Q.; Yao, L.; Qin, P.; Xu, J.; Sun, X.; Yao, B.; Ren, F.; Chen, W. Time-resolved fluorescent lateral flow strip for easy and rapid quality control of edible oil. *Food Chem.* **2021**, *357*, 129739, https://doi.org/10.1016/j.foodchem.2021.129739

5. Wu, Y.; Liu, J.; Yu, J.; Zhuang, J.; Ma, F.; Tan, J.; Shen, Z. A monoclonal antibody for identifying capsaicin congeners in illegal cooking oil and its applications. *Talanta* **2022**, *250*, 123686, https://doi.org/10.1016/j.talanta.2022.123686

6. Zhang, J.; Ma, F.; Zha, C.; Yang, Q.; Zhang, Q.; Zhang, W.; Li, P.; Sun, X. Effect of hapten structures on development of novel antibody against capsaicin and dihydrocapsaicin. *Chin. J. Anal. Chem.* **2022**, *50*, 100134, https://doi.org/10.1016/j.cjac.2022.100134

7. Zhang, J.; Zhang, M.; Yang, Q.; Wei, L.; Yuan, B.; Pang, C.; Zhang, Y.; Sun, X.; Guo, Y. A simple and rapid homogeneous fluorescence polarization immunoassay for rapid identification of gutter cooking oil by

detecting capsaicinoids. *Anal. Bioanal. Chem.* **2022**, *414*, 6127-6137, http://dx.doi.org/10.1007/s00216-022-04177-2

8. Yuan, D.; Li, S.; Zhang, L.; Ma, F.; Wang, H.; Zhang, Q.; Li, P. Rapid and sensitive quantification of capsaicinoids for edible oil adulteration by immunomagnetic solid-phase extraction coupled with time-resolved fluorescent immunochromatographic assay. *Food Chem.* **2023**, *404*, 134552, https://doi.org/10.1016/j.foodchem.2022.134552
9. Nie, K.; Zhang, J.; Xu, H.; Ren, K.; Yu, C.; Zhang, Q.; Li, F.; Yang, Q. Reverse design of haptens based on antigen spatial conformation to prepare anti-capsaicinoids&gingerols antibodies for monitoring of gutter cooking oil. *Food Chem.: X* **2024**, *22*, 101273, https://doi.org/10.1016/j.fochx.2024.101273
10. Jin, Z.; Sheng, W.; Sun, M.; Bai, D.; Ren, L.; Wang, S.; Wang, Z.; Tang, X.; Ya, T. Preparation of a capsaicinoids broad spectrum antibody and its application in non-enzyme immunoassay based on DMSNs@PDA@Pt. *J. Hazard. Mater.* **2024**, *466*, 133670, https://doi.org/10.1016/j.jhazmat.2024.133670
